# Chemical Composition and Content of Biologically Active Substances Found in *Cotinus coggygria, Dactylorhiza maculata, Platanthera chlorantha* Growing in Various Territories

**DOI:** 10.3390/plants10122806

**Published:** 2021-12-18

**Authors:** Stanislav Sukhikh, Lyudmila Asyakina, Maxim Korobenkov, Liubov Skrypnik, Artem Pungin, Svetlana Ivanova, Timothy Larichev, Viktoria Larina, Olesia Krol, Elena Ulrikh, Evgeny Chupakhin, Olga Babich

**Affiliations:** 1 Institute of Living Systems, Immanuel Kant Baltic Federal University, A. Nevskogo Street 14, 236016 Kaliningrad, Russia; SSukhikh@kantiana.ru (S.S.); LSkrypnik@kantiana.ru (L.S.); apungin@kantiana.ru (A.P.); surinac@mail.ru (V.L.); ole-jolie@yandex.ru (O.K.); chupakhinevgen@gmail.com (E.C.); olich.43@mail.ru (O.B.); 2 International Research Center “X-ray Coherent Optics”, Immanuel Kant Baltic Federal University, A. Nevskogo Street 14, 236016 Kaliningrad, Russia; alk_kem@mail.ru (L.A.); korobenkovmv@gmail.com (M.K.); 3 Department of Bionanotechnology, Kemerovo State University, Krasnaya Street 6, 650043 Kemerovo, Russia; 4 Natural Nutraceutical Biotesting Laboratory, Kemerovo State University, Krasnaya Street 6, 650043 Kemerovo, Russia; 5 Department of General Mathematics and Informatics, Kemerovo State University, Krasnaya Street 6, 650043 Kemerovo, Russia; 6 Department of Fundamental and Applied Chemistry, Kemerovo State University, Krasnaya Street, 6, 650043 Kemerovo, Russia; t-larichev@yandex.ru; 7 Institute of Agroengineering and Food System, Kaliningrad State Technical University, Soviet Avenue, 1, 236022 Kaliningrad, Russia; elen.ulrich@mail.ru

**Keywords:** *Cotinus coggygria*, *Dactylorhiza maculata*, *Platanthera chlorantha*, chemical composition, organic acids, vitamins

## Abstract

Medicinal plants (*Cotinus coggygria*, *Dactylorhiza maculata*, *Platanthera chlorantha*) growing in various territories (Kaliningrad, Moscow, and Minsk regions) were the objects of research. This paper presents a study of the chemical composition of these plants. To analyze the qualitative and quantitative composition of biologically active substances, the method of high-performance liquid chromatography was used. Atomic absorption spectrometry was used to study the content of trace elements. The content of organic acids and vitamins was determined by capillary electrophoresis using the Kapel-105/105M capillary electrophoresis system with high negative polarity. Extracts of medicinal plants were obtained on a Soxhlet apparatus using 70% ethanol as an extractant. It was found that among the biologically active substances in the plants under discussion, hyperoside, rutin (*C. coggygria*), Ferulic acid and Gallic acid (*D. maculata*), triene hydrocarbon (3,7-Dimethyl-1,3,6-octatriene), unsaturated alcohol (3,7-Dimethyl-2,6-octadien-1-ol), and benzyl acetate (*P. chlorantha*) prevailed. Samples of these medicinal plants contained trace elements (phosphorus, potassium, calcium, sodium, magnesium, and sulfur) and many aliphatic organic acids (succinic acid, benzoic acid, fumaric acid, citric acid, oxalic acid, and tartaric acid). The largest amount of biologically active substances and secondary metabolites of the studied plants from the Eastern Baltic is associated with climatic and ecological differences from other regions. The composition of these plants determines the potential of their use in feed additives for livestock and poultry as part of measures to improve the quality of livestock products. The use of medicinal plants for the production of feed additives is relevant in terms of improving regional economies, as well as improving the quality of life and nation’s health by providing ecologically clean livestock products.

## 1. Introduction

It is known that the *Cotinus coggygria* biomass is used as a medicinal raw material. Medicinal raw materials are being procured during the period from June to August [1,2]. *C. coggygria* is most valued for its secondary metabolites—hydrolyzable tannins, the content of which varies from 6 to 30% depending on the time of collection and the amount of sun absorbed by the leaf [3,4]. Secondary metabolites have astringent, anti-inflammatory, and antiseptic properties [5]. Other plant parts are also of interest; for example, the root-based decoction has antipyretic properties. Water and alcohol extracts from the shrub woody tissue have bactericidal properties. Decoctions of various this plant parts are used to treat stomatitis and pharyngitis. It is also worth mentioning that the *C. coggygria* leaves are included in the pharmacopeia [6,7].

*C. coggygria* extracts have high antioxidant activity in reaction with DPPH (IC_50_ = 2.6 ± 0.4 and 3.8 ± 0.5 μg/mL, respectively) and can slow down and completely inhibit the plant lipid peroxidation [8]. Additionally, a plant extract from the *Cotinus coggygria* leaves and flowers has anti-cancer and antigenotoxic properties. Acetone, methanol, water, ethyl acetate, and hexane extracts of this plant have antibacterial properties. An aqueous extract from this plant leaves has hepatoprotective properties [9].

It is known that *Dactylorhiza maculata* is mainly found in the northern and central regions of the non-chernozem zone [10]. The medicinal plant biomass contains mainly rutin, quercetin, ferulic acid, hexahydroxyflavonone, and gallic acid. In addition, the medicinal plant composition includes essential oils and bitter substances. This medicinal plant is used to treat gastritis, gastrointestinal tract diseases, and colic, and also as a strengthening agent for gastrointestinal tract disorders and urinary bladder diseases. In folk medicine, *D. maculata* is used as a general tonic with anti-inflammatory and enveloping properties [4]. 

*D. maculata* grows in coniferous, deciduous, and mixed forests, usually on moist and acidic soil, often in forested wetlands, sometimes enters swamps, and can also be found in dry areas. These plants can be found in the European part of Russia within the northern half of the forest zone and the Eastern Baltic, as well as outside Russia—in Western Europe (except for the southern regions) [11]. The plant propagates mainly by seeds and seed production is very high—the fruit contains up to 6200 seeds, totaling up to 186 thousand seeds per plant. Flowers are pollinated by flies, bees, bumblebees, and beetles. Up to 60% of flowers form fruits. The roots of the plant have mycorrhiza, which is especially intensively developed in young plants and much weaker in generative plants. In the Eastern Baltic region, the abundance of *D. maculata* is significant [4]. In Europe and adjacent territories, 18 species of this plant have been identified. Most authors agree with the division of the group into two subgroups according to the number of chromosomes, which is accompanied by a tendency towards morphological and ecological differentiation, which is not clearly expressed in all cases. *D. maculata* accumulates rutin, hyperoside, ferulic acid, quercetin, kaempferol, disulfuretin, sulfuretin, sulfurein, pentagalloyl glucose, and other valuable biologically active substances [5,11,12].

*Platanthera chlorantha* [10] is used as a drug for diseases of the gastrointestinal tract, bladder and also has bactericidal properties as it contains santolina triene, lilac alcohol, and aldehyde. *Platanthera bifolia* and *P. chlorantha* are widespread in the European part of Russia and the Caucasus [5]. 

*P. chlorantha* is rare in Russia—it can only be found in the Eastern Baltic. It takes this plant a long time to start blooming—more than five years will pass from the entry of a seed into the ground to the appearance of a flower, and plant reproduces mainly by seeds [6]. The flowers are organized into a sparse spike of up to 25 cm in length; *P. chlorantha* is bigger than *P. bifolia.* It blooms from late May to June. The stem is faceted, up to 60 cm high. The stem diameter is 4–5 mm [6]. *P. chlorantha* accumulates carene, santolina triene, benzyl acetate, lilac alcohol, lilac aldehyde, and others.

Plant-based feed additives are now widely used to treat and prevent many diseases of livestock and poultry. Their range is expanding annually, and the number of phytopreparations is increasing. The advantages of phytopreparations for livestock and poultry over synthetic drugs are their mild action and low toxicity.

A limiting factor in introducing domestic medicinal plants into the production of feed additives is the lack of information on the chemical composition of medicinal plant raw materials and insufficient knowledge of the medicinal properties of plant preparations [6]. This problem can be solved by studying the chemical composition and content of BAS of medicinal plants and searching for promising methods for their extraction and purification for use as feed additives with antibiotic properties. The use of medicinal plants to produce feed additives is relevant in improving regional economies and improving the quality of life and the nation’s health by providing ecologically clean livestock products.

Medicinal plants and plant extracts of a complex of biologically active substances can be a promising raw material for the production of antimicrobial components [13,14]. Biological activity depends on the qualitative and quantitative composition of the secondary metabolites of the medicinal plant, which is not constant [15,16,17]. The content of secondary metabolites depends on the climatic conditions of the region, the chemical composition of the soil in which the medicinal plant grows, as well as on the ecological situation in the area of cultivation of the medicinal plant.

Until recently, studies of medicinal plants *C. coggygria*, *D. maculata*, and *P. chlorantha* on the territory of the Russian Feleration were carried out with a narrow ecological-phytocenotic amplitude, in habitats of the same type, mainly on sedge-sphagnum peats, swamped shrub-sphagnum, and sphagnum pine forests. It was found that these plants form rather large full-member cenopopulations, with a predominance of generative and immature plants. It was shown that the size of these species varies depending on the location. A gradual decrease in the habitus of plants from the southern part of Russia to the north was noted, while an increase in the size of flowers was noted in the mountains. The pollination efficiency (57.4%) and seed productivity (2835 seeds on averege, more than 90% of which were full-value) were studied [5].

The stability of cenopopulations of medicinal plants *C. coggygria, D. maculata*, and *P. chlorantha* was studied. It was proved that the stability of cenopopulations is maintained due to the long duration of the generative period and irregular (once every several years) mass development of juveniles. The chemical composition, physicochemical, and biological properties of these plants growing in the territory of the Eastern Baltic, Moscow, and Minsk regions have not been studied much, and they have not been compared with each other [5].

Since all three studied medicinal plants have significant antimicrobial and antioxidant activity, as well as a balanced chemical composition, it is proposed to use them together to produce a multifunctional feed additive for livestock and poultry. It is planned to partially replace feed antibiotics with this feed additive based on the extracts of medicinal plants, which can have a positive effect on the economically useful qualities of livestock and poultry [5].

Medicinal plants can be beneficial not only for humans but also for animals and birds. The properties and characteristics of plants are set by the components they contain, which are determined by both climatic and geographical conditions. The mild climate of the Eastern Baltic region and the characteristics of the mentioned medicinal plants suggest the possibility of their application in feed additives for livestock and poultry. Since this possibility has not been previously investigated, this study aimed to determine the chemical composition and content of biologically active substances in the Eastern Baltic medicinal plants (*C. coggygria*, *D. maculata*, *P. chlorantha*) and compare them with medicinal plants growing in other territories.

## 2. Results

The results of studying the chemical composition of medicinal plant samples are presented in Table 1.

Further, the qualitative and quantitative composition of biologically active substances in medicinal plants were analyzed [18,19,20,21]. The results of studying the qualitative and quantitative composition of medicinal plants growing in different regions are presented in Table 2, Table 3 and Table 4 and Appendix A.

Studying the vitamin and microelement composition of medicinal plants growing in the Kaliningrad region and in different regions, as well as their organic acid content, is also of interest (Table 5, Table 6, Table 7 and Table 8).

The results of studying the biological (antimicrobial and antioxidant) activity of the investigated plant species extracts from different regions are presented in Table 9.

**Figure 1:**
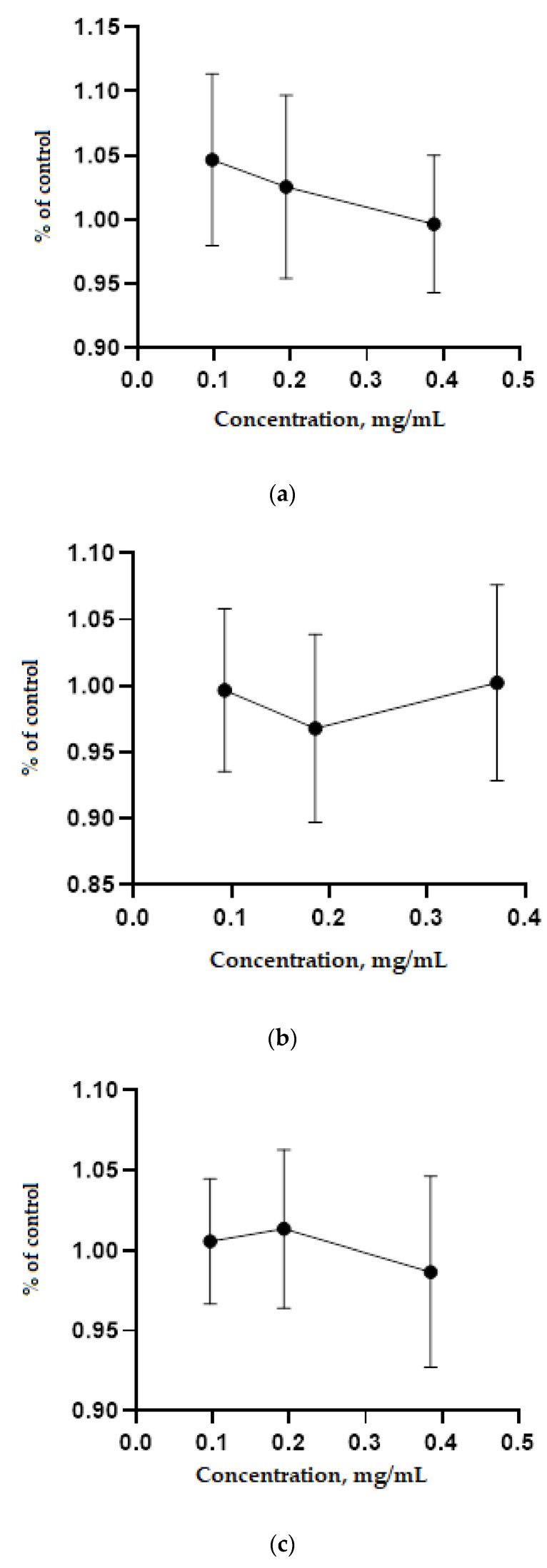
Determination of the dose-dependent effect of the toxicity of (**a**) *C. coggygria*, (**b**) *D. maculate*, and (**c**) *P. chlorantha* extracts on the HEK293 cell line after 48 h of incubation.

Figure 1 shows the results of determining the dose-dependent effect of the toxicity of plant extracts.

## 3. Discussion

The results presented in Table 1 show that the studied samples of the investigated plants species from different regions are rich in protein substances, lipid fraction, and fiber. 

The research results indicate that the mass fraction of protein in these plants varied in the ranges from 11.91% to 12.30%, from 8.02% to 8.28%, and from 9.13% to 9.49%, respectively. The fat content in the studied plant samples ranged from 1.00% to 1.21%, from 2.10 to 2.23%, and from 1.14 to 1.34%. The highest fiber content was found in *P. chlorantha* from the Eastern Baltics, and the lowest in *P. chlorantha* from the Moscow region. The minimal fiber content was found in *Cotinus coggygria* growing in the Moscow region, and the maximal in *C. coggygria* growing in the Eastern Baltics. There were no significant differences in the content of the considered component in the samples of plants from different growing regions, but such differences were observed for plant species. Analysis of the chemical composition of the studied plants using standard reference methods by Egorova and Startseva [22] showed that *P. chlorantha* has the highest ash content 17.30 mg/kg and moisture content 89.40 mg/kg, while *D. maculata* has the lowest ash content 12.04 and moisture content 68.40. *P. chlorantha* and *C. coggygria* had the highest content of protein 30.02 mg/kg and 32.13 mg/kg, respectively, and carbohydrates 54.00 mg/kg and 56.06 mg/kg, respectively, while *D. maculata* had the lowest content of protein 25.06 mg/kg and carbohydrates 40.23 mg/kg. *P. chlorantha* has the highest crude fiber content 25.05 mg/kg, while *D. maculata* has the highest crude fat content 18.22 mg/kg. The data presented in [22] described the studied medicinal plants grown in vitro, therefore they had a constant, reproducible composition and properties, such plants are protected from adverse climatic conditions, receive an equal amount of fertilizing, moisture, and light. Plants grown in vitro are not subject to damage by pests: insects, molds, bacterial rot. Considering the above reasons, the plants we studied are somewhat different from those grown in vitro. The composition and properties of intact plants vary depending on many environmental factors.

Polyphenolic compounds, organic acids, glycosides, alcohols, aldehydes, which have potent antioxidant, antimicrobial, anti-inflammatory, immunomodulatory, and anti-mutagenic properties, have been isolated from the studied medicinal plants, growing in the studied regions (Table 2, Table 3 and Table 4). Analysis of the results found no significant differences in the composition of biologically active components in plant samples from different regions. The largest amount of biologically active substances was found in the investigeted plants species growing in the territory of the Eastern Baltic, and the smallest in the medicinal plants growing in the Moscow region.

The increased content of biologically active substances in medicinal plants of the Eastern Baltics can be explained by the mild climate and landscape diversity, favorable for the growth of these plants and the accumulation of the maximum amount of biologically active substances. The Baltics are located in the Baltic Sea area, where the climate is quite mild, moderately maritime, and continental. In winter, frosts are negligible. Summers are warm but rainy. The coastal areas are slightly milder but also more humid and windy, while the eastern part has a slightly more continental climate. Belarus, as with the Eastern Baltics, is located in the Baltic artesian basin in the northwest of the East European platform; however, the climate in the Minsk region is cold and temperate. Minsk region has cold summers and significant rainfall, even in dry months. The Moscow region, as a developed industrial region, is characterized by a temperate continental climate, with a strong influence of the Atlantic maritime, with a pronounced seasonality. Severe frosts are frequent and usually long. The absence of large bodies of water contributes to rather large fluctuations in temperature and the influence of the Gulf Stream, caused by Atlantic and Mediterranean cyclones, provide relatively high levels of atmospheric precipitation.

The study [22] demonstrated that *C. coggygria* contains 1.03% myricetin with a molecular weight of 318, 1.41% fisetin with a molecular weight of 303, 1.40% rutin with a molecular weight of 612, 0.09%, quercetin with a molecular weight of 338, and is 0.08% flavonoid with unknown structure [8]. The total percentage of *D. maculata* flavonoids was determined as 4.01% [7], and *P. chlorantha* 5.6%.

The results (Table 5) indicate that the studied medical plants contain a large number of mineral elements, among which phosphorus, potassium, calcium, sodium, magnesium, and sulfur should be noted.

When studying the investigated plant species, Egorova and Startseva [22] found the highest Na content in *D. maculata* (3.649%) and *C. coggygria* (3.25%). *P. chlorantha* contained 1.279 ± 0.003 mg/L of Na. The potassium (K) content in plants ranged from 1024 mg/mL in *C. coggygria* to 1618 mg/mL in *D. maculata*, and the average K content in *P. chlorantha* was ±600 mg/mL. These plants, as with the ones we studied, are considered good sources of K; their K values are much higher than the minimum recommended daily intake of K, which is 3500 mg. The highest calcium (Ca) concentration is found in such plants as *C. coggygria* (13.09%), followed by *P. chlorantha* (6.13%) and *D. maculata* (6.6%) [23]. The analysis of the results, presented in Table 4, indicates that the studied samples of medicinal plants contain a large amount of aliphatic organic acids, such as succinic acid, benzoic acid, fumaric acid, citric acid, oxalic acid, and tartaric acid [21,22,24]. Hassan et al. [23] showed that *C. coggygria* is characterized by low oxalic acid content. Thus, the oxalic acid content in *Cotinus coggygria* [8] in our studies is 3.74 times lower than its content in *D. maculata* [7].

Analysis of the results presented in Table 6, Table 7 and Table 8 shows that the medicinal plants, growing in the studied regions, contain B vitamins (thiamine and riboflavin) and ascorbic acid (vitamin C) [23,25]. Egorova and Startseva [22] showed that the accumulation of ascorbic acid in *C. coggygria* was 70.36 mg/kg, which correlates well with our studies. As for the content of organic acids and vitamins, the data obtained in our studies are in good agreement with the data presented in [22,26].

The chemical and spatial structures of secondary metabolites of medicinal plants are comparable to the metabolic products of microorganisms, which contributes to the fact that plant extracts of the BAS complex are more actively involved in biological processes [8]. In this regard, the biological activity (antioxidant and antimicrobial) of the complex of biologically active substances from the studied medicinal plants growing in the territory of the Eastern Baltic was studied.

It was shown that samples of BAS complex extracts isolated from the studied medicinal plants from different regions (Table 9) had an inhibitory effect on the *E. coli* test strain. Lysis zone was from 13.6 to 16.5 mm, from 15.2 to 17.0 mm, and from 15.1 to 17.0 mm for *C. coggygria* [8], *D. maculata* [7], and *P. chlorantha*, respectively. *Platanthera chlorantha* samples from the Eastern Baltics exhibited high antibacterial activity against *S. aureus* (lysis zone 18.0 mm).

The growth of the opportunistic bacterium *L. mesenteroides* was negatively influenced by samples of *D. maculata* [7] and *P. chlorantha* extracts from the Eastern Baltics, while the lowest activity was demonstrated by the plants from the Moscow region. The *P. chlorantha,* growing in the Eastern Baltics, components inhibited the growth of the *P. vulgaris* strain (the diameter of the lysis zone was 16.5 mm).

All tested medicinal plants growing in different regions had a high antioxidant status (the antioxidant activity of the samples varied from 107.29 mg AA/g to 220.43 mg AA/g) (Table 9). The minimum antioxidant activity was recorded for *Cotinus coggygria* samples, growing in the Moscow region, (145.09 mg AA/g) [8], the maximum—for *P. chlorantha* samples, growing in the Eastern Baltics, (220.43 mg AA/g). The biological activity of *D. maculata* and *P. chlorantha* extracts also depended on the climatic conditions of the studied regions and was directly related to the accumulation and activity of biologically active substances in these plants [7]. The study [26] confirmed that climatic and environmental factors significantly affect the accumulation of biologically active substances in plants. The main environmental factors affecting BAS include average annual precipitation, average July temperature, frost-free period, length of solar day, soil pH, soil organic matter, and readily available potassium in the soil. The average annual precipitation is the most important determining factor, which significantly and negatively correlates with the content of biologically active substances (*p* < 0.001). Climate factors have a greater effect on the content of biologically active substances than the composition of the soil. Ecological and climatic factors, in terms of the content of secondary metabolites and their activity, are different for different places of growth.

The antimicrobial and antioxidant activities of the studied medicinal plants are confirmed by the studies carried out in [27]. In this study, the antimicrobial activity of young shoots of *C. coggygria* was studied. Acetone extract and a derivative fraction of ethyl acetate effectively suppressed the growth of gram-positive and gram-negative bacteria, while the chloroform fraction exhibited pronounced activity against the yeast *Candida albicans*. The ethyl acetate fraction showed a significant ability to reduce iron, very high activity in scavenging DPPH radicals, and inhibition of lipid peroxidation, which proves antioxidant activity of the faction. High amounts of total phenols, tannins, and flavonoids were detected in the ethyl acetate fraction, which also had significant anti-inflammatory and cytotoxic effects [2].

The extracts of the studied medicinal plants did not have a toxic effect on the HEK293 cell line in the concentration range from 0.1 to 0.4 mg/mL (Figure 1), which demonstrates that these plants can be safely used as part of feed additives for livestock and poultry.

The compositions of the extracts given in [4,22,23,28] differ from the extracts in our previous [7,8] and these studies, because they described the different habitats and collection areas of the studied medicinal plants. The composition of the extracts is influenced by both climatic factors (precipitation, winds, summer and winter temperatures, pressure, influence of cyclones and remoteness from the sea), and the terrain, fertility, reclamation, soil erosion, application of organic and mineral fertilizers, pests (insects, molds, bacteria). It is also influenced by the parameters and modes of extraction: extraction temperature, extractant concentration, extraction time, methods and temperature of drying and grinding the extracts, and conditions of their storage. The determination of the composition of extracts is partially influenced by the process of sample preparation of the extract before chromatographic and electrophoretic studies, human factors, and many other factors.

## 4. Materials and Methods

### 4.1. Objects of Research

**Figure 2:**
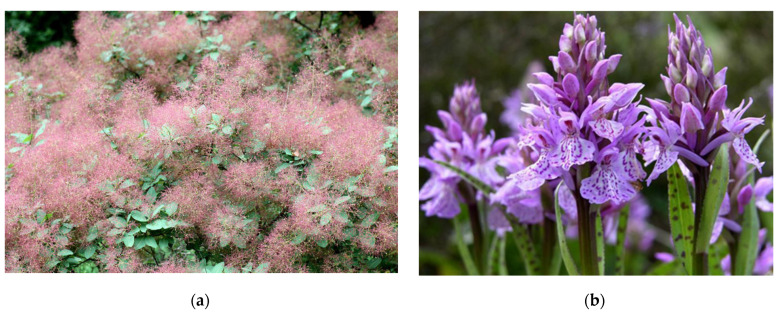

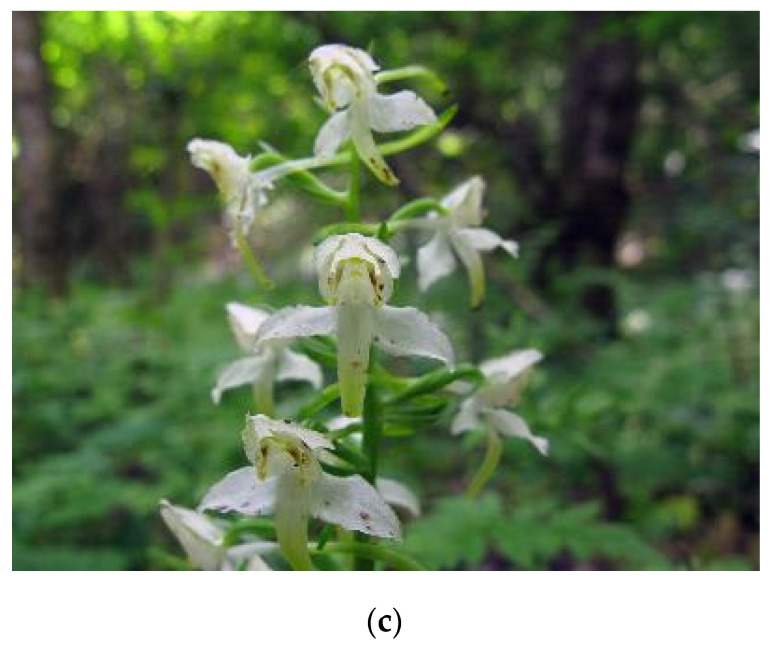
Medicinal plants selected for research: (**a**) *C. coggygria*; (**b**) *D. maculata,* and (**c**) *P. chlorantha*. The photos were selected from the IKBFU collection.

Medicinal plants (*Cotinus coggygria*, *Dactylorhiza maculata*, *Platanthera chlorantha*) of the Kaliningrad, Moscow, and Minsk regions were selected as objects of research (Figure 2). The species biomaterial was confirmed by Pungin A.V., the head of the herbarium of the Institute of Living Systems of the Immanuel Kant Baltic Federal University (protocol No. 6-8/2020). Aerial parts of medicinal plants were collected.

Mature plants were selected to analyze the chemical composition [8]. The ratio of shoots/leaves/flowers in the harvested biomass was on average 4:2:1 for each medicinal plant. During the research, the chemical composition of the whole medicinal plant was studied.

### 4.2. Determination of the Chemical Composition of Medicinal Plants

The mass fraction of crude protein, mass fraction of fiber, mass fraction of fat (crude fat), mass fraction of ash, cellulose, as well as the content of biologically active substances were selected as the leading indicators characterizing the chemical composition of medicinal plants [9,10,11].

Crude protein content, mass fraction of fiber, and crude fat were evaluated according to GOST 32040-2012. The essence of the method consists in measuring the intensity of radiation reflected from the analyzed sample in the near-infrared region of the spectrum, determining the content of crude protein, crude fiber, crude fat, and moisture according to calibration equations obtained from the results of measurements of the intensity of reflected radiation from samples with known values of the determined parameters established by the standard chemical methods. Infrascan-3150 analyzer (Ekan, St. Petersburg, Russia) was used to measure the intensity of radiation reflected from the analyzed sample in the near-infrared region (from 800 to 2500 nm) with the indication of the results on a personal computer screen or the analyzer display. Measurements are performed immediately after filling the cuvette. Each portion of the analyzed sample is loaded into the analyzer once.

The mass fraction of crude ash was determined following GOST 32933-2014 (ISO 5984:2002). The essence of the method lies in the ashing of organic substances of the analyzed sample by calcining and weighing the resulting residue. The pan with a weighed sample is gradually heated on an electric stove or over a gas burner until the sample is charred. The pan is transferred to a muffle furnace, preheated to a temperature of 550 °C, and left for 3 h. After cooling, the pan is quickly weighed on a balance with an accuracy of ±0.001 g. To determine the mass fraction of cellulose, GOST 16932-93 (ISO 638-78) was used. The method is used for wet or air-dry cellulose samples that do not contain substances other than water that volatilize at the specified drying temperature. The method is used for samples taken for chemical and physical analyzes in laboratory conditions when it is simultaneously required to determine the dry matter content. 10 g of cellulose is weighed correct to three places of decimals in a closed, pre-dried, and weighed vessel. To study the chemical composition, all studied samples were cut with scissors to a size of 0.5–1.0 cm. All chemicals (analytical or higher grade) used in this study were purchased from Fluka/Sigma-Aldrich (Sigma-Aldrich Rus, Moscow, Russia).

### 4.3. Determination of Biologically Active Substances of Medicinal Plants

General laboratory and analytical equipment was used: AND HR-202 i analytical balances (A&D, Tokyo, Japan), CAS CUW420H balances (CAS Corporation Ltd., Gwangju, Korea), Kitfort KT-107 electric stove (Kitfort, Moscow, Russia), Binder FDL115 drying oven (BINDER GmbH, Tuttlingen, Germany), laboratory mill (IKA, Staufen, Germany), SNOL 6/1 laboratory chamber resistance electric furnace (Umega, Vilnius, Lithuania), testing screen (Novolab, Novosibirsk, Russia), automatic potentiometric titrator ATP-02 (Akvilon, St. Petersburg, Russia), rotary evaporator IKA RV 8 V (IKA, Staufen, Germany), water treatment system Elix 5 UV (Millipore, Molsheim, France), water bath-thermostat with stirring WB-4MS (BioSan, Riga, Latvia), potential hydrogen meter for aqueous solutions pH meter ST3100-F (Ohaus, Parsippany, NJ, USA), magnetic stirrer MS-01 (ELMI, Latvia), portable multifunctional scales SPS2001F (Ohaus, Newark, NJ, USA), bidistiller TX 25-11.15.92-81 BS.W39 (Khimlabpribor, Moscow, Russia).

To analyze the qualitative and quantitative composition of biologically active substances in medicinal plants, the method of high-performance liquid chromatography (HPLC) and a Shimadzu LC-20AD chromatograph (Shimadzu, Kyoto, Japan) were used according to the General Pharmacopoeia Monograph 1.2.1.2.0005.15. Detection was carried out using a diode array detector in the detection range of 180 nm–900 nm (rutin at 375 nm, hyperoside at 410 nm, ferulic acid at 307 nm, quercetin at 343 nm, kaempferol at 410 nm, disulfuretin at 595 nm, sulfurein at 410 nm, sulfurein at 510 nm, gallic acid at 270 nm, methyl gallate at 385 nm, pentagalloyl glucose at 740 nm, 3,3′,4′, 5,6,7-hexahydroxyflavonone at 331 nm, 3,3′,4′,5,5′,7-hexahydroxyflavonone at 300 nm, 3-O;-α-L-rhamnofuranoside at 339 nm, 3,3′,4′,5.5′,7-hexahydroxyflavulium(1+) at 254 nm, 7-O;-β-D glucopyranoside at 566 nm, 3,3′,4′,7-tetrahydroxyflavonone at 430 nm, 3-carene at 313 nm, santolinatriene at 416 nm, 1,2-hexanediol-2-benzoate at 590 nm, 3,7-dimethyl-1,3,6-octatriene 326 nm, 3,7-dimethyl-2,6-octadien-1-ol at 516 nm, benzyl acetate at 595 nm, lilac alcohol at 316 nm, and lilac aldehyde at 330 nm), the flow rate of the eluent in all cases was 1 mL/min, elution was carried out in a gradient mode, the time and gradient were selected individually for each separation, the mixture of treated water (MQ purification level) and acetonitrile with the addition of 0.1% trifluoroacetic acid was used as solvents, and separation was carried out on a reversed-phase Phenomenex column (Torrance, California, USA) 250 × 2.5 mm, particle size 10 μm, sorbent silica gel modified C-18, with phenyl end-capping. All chemicals (analytical or higher grade) used in this study were purchased from Fluka/Sigma-Aldrich (Sigma-Aldrich Rus, Moscow, Russia).

1,2-hexanediol-2-benzoate (certified reference material, CAS:23246-47-1), 3,3′,4′,5,5′,7-hexahydroxyflavulium(1+) (certified reference material, CAS:529-44-2), 3,3′,4′,5,6,7-hexahydroxyflavonone (certified reference material, CAS:90-18-6), 3,3′,4′,7-tetrahydroxyflavonone (certified reference material, CAS:528-48-3), 3,3′,4′,5,5′,7-hexahydroxyflavonone-3-*O*-glycoside (certified reference material, CAS:153-18-4), 3,7-dimethyl-1,3,6-octatriene (certified reference material, CAS:13877-91-3), 3,7-dimethyl-2,6-octadien-1-ol (certified reference material CAS:624-15-7), 3-Carene (certified reference material, CAS:13466-78-9), 3-*О-*α-L-rhamnofuranoside (certified reference material, CAS:63864-94-8), 7-*О*-β-D glucopyranoside benzyl acetate (certified reference material, CAS:10343-13-2), disulfuretin (certified reference material, CAS:97-77-8), ferulic acid (≥99%, 128708), gallic acid (≥ 97.5%, G7384), hyperoside (certified reference material, CAS:482-36-0), kaempferol (≥97.0%, 60010), methyl gallate (certified reference material, CAS:99-24-1), pentagalloyl glucose (certified reference material, CAS:14937-32-7), quercetin (≥95.0%, Q4951), santolina triene, ruthin (≥95.0%, PHL89270), sulfurein (certified reference material, CAS:120-05-8), sulfuretin (certified reference material, CAS: 120-05-8), lilac alcohol (CAS: 33081-36-6) and lilac aldehyde (certified reference material, CAS: 51685-39-3) were purchased from Fluka/Sigma-Aldrich (Sigma-Aldrich Rus., Moscow, Russia). The values of the standard samples for BAS were used to construct calibration curves, which were used to determine the number of components.

### 4.4. Determination of the Content of Trace Elements in Medicinal Plants

Method of atomic absorption spectrometry using Shimadzu atomic absorption spectrometer (Lumex, St. Petersburg, Russia) was used to study the content of trace elements in medicinal plants being the object of the study. For this, concentrated nitric acid in an amount of 10 mL was added to the medicinal plant ground in a laboratory mill (IKA, Germany), and the sample was decomposed in a MARS-6 microwave oven (DAF Trucks, Amsterdam, the Netherlands) for several hours. Upon completion of decomposition, the sample was filtered through a paper filter and diluted with an aqueous nitric acid solution (the acid concentration did not exceed 1%). Next, measurements were carried out using a Shimadzu atomic absorption spectrometer (Kyoto, Japan).

### 4.5. Determination of the Content of Organic Acids in Medicinal Plants

The content of organic acids in medicinal plants was determined by capillary electrophoresis using the Kapel-105/105M capillary electrophoresis system (Lumex, St. Petersburg, Russia) with high negative polarity. The method is based on extracting acids from the test sample with distilled water, separation, and quantitative determination of components by capillary electrophoresis (CE) with indirect detection at a wavelength of 190 nm.

### 4.6. Determination of the Content of Vitamins in Medicinal Plants

The vitamin composition of medicinal plants was assessed by the method of capillary electrophoresis using the Kapel-105/105M capillary electrophoresis system with the negative polarity of high voltage. The method is based on the migration and separation of the ionic forms of the analyzed components under the action of an electric field due to their different electrophoretic mobility, followed by registration at a wavelength of 200 nm.

To avoid errors and minimize errors, each experiment was repeated three times. The average of three repetitions was considered as a result [12].

### 4.7. Determination of the Biological (Antimicrobial) Activity of Plant Extracts

Extracts of medicinal plants were produced to determine the biological activity. Extraction was carried out on a Soxhlet apparatus (Velp Scientifica, Usmate, Italy) using 70% ethanol as an extractant [29].

The antimicrobial activity of medicinal plant extracts was determined in terms of the growth of opportunistic and pathogenic test strains of microorganisms by the diffusion method (on a solid nutrient medium).

The following medical and natural test strains were used as opportunistic and pathogenic microorganisms: *E. coli* ATCC 25922, *S. aureus* ATCC 25923, *P. vulgaris* ATCC 63, *C. albicans* EMTC 34, *L. mesenteroides* EMTC 1865). Commercially available reagents of domestic and foreign production with a reagent-grade purity and higher were used. To study antimicrobial activity, the following laboratory equipment was used: laminar flow cabinet class 2/type A BAVp-01-“Laminar-S”-1.5 (Laminarnyye systemy, Moscow, Russia), thermostat Shaking Incubator LSI-3016A (Daihan Labtech, Namyangju, North Korea), autoclave DGM-80 (Pharma Apparate Handel AG, Zug, Switzerland), direct microscope AxioScope A1 (Zeiss, Jena, Germany).

*E. coli* strain was cultivated on a dense LB nutrient medium at 37 °C. *S. aureus* strain was cultured on milk salt agar using a feed yeast hydrolyzate in an amount of 24.0 g, NaCl 75.0 g. The cultivation process was carried out at 36 °C. The opportunistic bacterium *P. vulgaris* was cultivated in meat-peptone broth at a temperature of 36 °C. The cultivation of the microscopic fungus *C. albicans* was carried out in Sabouraud nutrient medium at 25 °C. The *L. mesenteroides* strain cultivated on a dense nutrient medium (yeast extract—4.0%, meat extract—10.0%, casein hydrolyzate—10.0%, glucose—20.0%, ammonium citrate—2.0% sodium acetate—5.0%, tween-80—1.0%, disubstituted potassium phosphate—2.0%, magnesium sulfate—0.2%, and manganese sulfate—0.05%). Cultivation was carried out at 36 °C.

The diffusion method for determining the antimicrobial activity of plant extracts consisted in the following. A lawn of test strain was produced by spreading it on an agar nutrient medium, and BAS complex plant extracts were placed on the lawn. A paper disc with a nutrient medium was used as a control, and a disc with antibiotic rifampicin (from a standard kit) was used as a reference drug. Petri dishes were incubated at a temperature corresponding to the optimal growth temperature for each test strain for 24.0 ± 0.5 h. The results considered the presence and size (mm) of the transparent zone of the absence of microorganism growth around the disc [30].

### 4.8. Determination of the Antioxidant Activity of Plant Extracts

The antioxidant activity of plants was determined by their ability to reduce the 2,2-diphenyl-1-picrylhydrazyl radical (DPPH, C18H12N5O6, M = 394.33). The reaction of the interaction of antioxidants with DPPH-radical proceeds as follows:

DPPH∙ + AH → DPPH − H + A∙

The reduction of the DPPH radical with an antioxidant results in a decrease of the intense purple-blue color of DPPH in ethanol. The reaction is monitored by the change in optical density by conventional spectrophotometric methods.

Plant extracts were mixed with 2.85 mL of a freshly prepared 0.1 mM solution of 2,2-diphenyl-1-picrylhydrazyl for analysis. The mixture was incubated in the dark at room temperature for 30 min. The decrease in optical density compared to the control (70% methanol solution) was recorded at 517 nm (UV-3600 spectrophotometer, Shimadzu, Kyoto, Japan) [27]. Solutions of ascorbic acid (AA) of known concentration were used as a standard solution. The analysis results were expressed in mg AA equivalent per gram of dry weight of plant extracts (mg AA/g). The analysis of the antioxidant activity of the samples was repeated three times.

### 4.9. In Vitro Study of the Toxicity of Medicinal Plant Extracts

MTT assay was used to study the toxivity of the plant extarcts. For this, HEK293 cells (Sputnik-K, Moscow, Russia), cell lines obtained from human embryonic kidneys, were planted in a 96-well plate in the amount of 104 cells per well. The tested plant extracts were added to the plate at 5 different concentrations: 0.05 mg/mL, 0.15 mg/mL, 0.3 mg/mL, 0.6 mg/mL, and 1 mg/mL. The buffer in which the extracts were dissolved was used as a control. Cells treated with extracts of medicinal plants were incubated for 72 h in a CO_2_ incubator, after which the medium with the extracts was replaced with a working MTT solution (5 mg/mL in PBS buffer), which consisted of 20 μL of the stock and 80 μL of the medium for cell growth. After 2 h in the incubator, the medium with MTT was replaced with 100 μL of lysis buffer (10% SDS in a 1:1 mixture of water:DMF, pH 4.7 adjusted with acetic acid) and incubated for 8 h until the formazan crystals were dissolved [29,31]. After that, the spectophotometer readings were taken at a wavelength of 570 nm and calculated by the Equation (1) [32]:

(1) $$V=\frac{\left(D_{s}-D_{g}\right)}{\left(D_{std}-D_{g}\right)}100\%,$$

where *V*—viability (cell viability relative to the control), %; *D_s_*—optical density of test wells (samples); *D_g_*—optical density of the medium; *D_std_*—optical density of control wells (samples).

### 4.10. Statistical Analysis

Each experiment was repeated three times, and the data are expressed as means ± standard deviation. Data processing was carried out via the standard methods of mathematical statistics. Post hoc analysis (Tukey test) was undertaken to identify samples that were significantly different from each other. The equality of the variances of the extracted samples was checked using the Levene test. The data were subjected to analysis of variance (ANOVA) using Statistica 10.0 (StatSoft Inc., 2007, Tusla, OK, USA). Differences between means were considered significant when the confidence interval was below 5% (*p* < 0.05).

## 5. Conclusions

The research resulted in the determination of the chemical composition and content of biologically active substances *C. coggygria*, *D. maculata*, and *P. chlorantha*, growing in various territories (Eastern Baltic (Russia), the Moscow region (Russia), and the Minsk region (Belarus)). It was proven that the investigated plant species contain a large number of mineral elements, among which phosphorus, potassium, calcium, sodium, magnesium, and sulfur should be noted. It was demonstrated that the studied samples of medicinal plants contain a large amount of aliphatic organic acids, such as succinic acid, benzoic acid, fumaric acid, citric acid, oxalic acid, and tartaric acid. The studied medicinal plants contain B vitamins (thiamine and riboflavin) and ascorbic acid (vitamin C). It was shown that the BAS complexes of the studied medical plants had an inhibitory effect on pathogenic and opportunistic microorganisms and high antioxidant status. The largest amount of biologically active substances and secondary metabolites were found in the studied plants growing in the Eastern Baltic, the smallest—the studied medicinal plants growing in the Moscow region. This BAS distribution is associated with the climatic and ecological characteristics of the regions where medicinal plants grow. The antimicrobial toxic effect of the extracts of the studied medicinal plants was investigated. It was found that all medicinal plants had a detrimental effect on pathogenic and opportunistic microflora and did not have a toxic effect on the HEK293 cell line.

Among the biologically active substances of the studied plant species, flavonoids were identified. These BAS are of interest for the production of feed additives for livestock, as they have potential to be an alternative to antibiotics. Plant-based feed additives are now widely used to treat and prevent many diseases in livestock and poultry. Their range is expanding every year and the number of herbal remedies is increasing. The advantages of phytopreparations for livestock and poultry over synthetic preparations are mild action and low toxicity. The revealed characteristics of the investigated plant species, growing in the territory of the Eastern Baltic, reveal their potential for use in the food and, especially, feed industry, as components with antibacterial and antioxidant properties [6,33,34].

## Figures and Tables

**Table 1:** Chemical composition of the studied medicinal plants growing in different regions.

Plant Samples	Growing Region	Mass Fraction %				
		Moisture	Crude Protein	Fiber	Crude Fat	Crude Ash
*C*. *coggygria*	I	9.82 ± 0.29	12.30 ± 0.36	10.44 ± 0.31	1.21 ± 0.03	5.40 ± 0.16
	II	9.05 ± 0.29	11.91 ± 0.36	10.11 ± 0.31	1.00 ± 0.03	5.11 ± 0.16
	III	9.24 ± 0.29	12.00 ± 0.36	10.22 ± 0.31	1.13 ± 0.03	5.27 ± 0.16
*D*. *maculate*	I	9.71 ± 0.28	8.28 ± 0.30	14.37 ± 0.43	2.23 ± 0.06	6.27 ± 0.18
	II	9.12 ± 0.28	8.02 ± 0.30	13.77 ± 0.43	2.10 ± 0.06	5.87 ± 0.18
	III	9.21 ± 0.28	8.10 ± 0.30	14.17 ± 0.43	2.16 ± 0.06	6.18 ± 0.18
*P*. *chlorantha*	I	15.88 ± 0.47	9.49 ± 0.28	18.85 ± 0.56	1.34 ± 0.03	7.82 ± 0.23
	II	14.68 ± 0.47	9.13 ± 0.28	17.96 ± 0.56	1.14 ± 0.03	6.98 ± 0.23
	III	15.16 ± 0.47	9.26 ± 0.28	18.34 ± 0.56	1.22 ± 0.03	7.37 ± 0.23

I—Eastern Baltics (Russia); II—Moscow region (Russia); III—Minsk region (Belarus). Values in columns (for each plant species) did not have significant differences (*p* > 0.05), as assessed by the post hoc test (Tukey test). Data presented as a mean ± SD (n = 3).

**Table 2:** Assay of individual biologically active substances (content, mg/kg) in *C. coggygria* samples from different regions.

Peak No.	Substances	Growing Region		
		I [8]	II	III
1	Rutin	46.33 ± 1.38 ^a^	35.39 ± 1.38 ^b^	41.15 ± 1.38 ^c^
2	Hyperoside	36.64 ± 1.09 ^a^	36.11 ± 1.09 ^a^	36.10 ± 1.09 ^a^
3	Ferulic acid	5.12 ± 0.15 ^a^	4.32 ± 0.15 ^b^	5.10 ± 0.15 ^a^
4	Quercetin	12.33 ± 0.39 ^a^	11.14 ± 0.39 ^a^	11.95 ± 0.39 ^a^
5	Kaempferol	12.25 ± 0.36 ^a^	11.47 ± 0.36 ^a^	11.97 ± 0.36 ^a^
6	Disulfuretin	0.210 ± 0.006 ^a^	0.110 ± 0.006 ^b^	0.191 ± 0.006 ^a^
7	Sulfuretin	0.99 ± 0.02 ^a^	0.14 ± 0.02 ^a^	0.77 ± 0.02 ^b^
8	Sulfurein	0.220 ± 0.006 ^a^	0.137 ± 0.006 ^b^	0.203 ± 0.006 ^a^
9	Gallic acid	3.82 ± 0.11 ^a^	3.00 ± 0.11 ^a^	3.17 ± 0.11 ^a^
10	Methyl gallate	2.98 ± 0.08 ^a^	2.25 ± 0.08 ^a^	2.71 ± 0.08 ^a^
11	Pentagalloyl glucose	17.64 ± 0.52 ^a^	15.38 ± 0.52 ^b^	16.45 ± 0.52 ^c^
12	3,3′,4′,5,6,7-hexahydroxyflavonone	12.84 ± 0.38 ^a^	11.03 ± 0.38 ^b^	11.86 ± 0.38 ^ab^
13	3,3′,4′,5,5′,7-hexahydroxyflavonone	12.36 ± 0.37 ^a^	10.88 ± 0.37 ^b^	11.72 ± 0.37 ^ab^
14	3-*О-*α-L-rhamnofuranoside	6.81 ± 0.20 ^a^	6.10 ± 0.20 ^a^	5.85 ± 0.20 ^a^
15	3,3′,4′,5,5′,7-hexahydroxyflavulium(1+)	4.37 ± 0.13 ^a^	3.13 ± 0.13 ^b^	3.31 ± 0.13 ^b^
16	7-*О*-β-D glucopyranoside	10.89 ± 0.32 ^a^	9.63 ± 0.32 ^a^	9.99 ± 0.32 ^a^
17	3,3′,4′,7-tetrahydroxyflavonone	6.72 ± 0.20 ^a^	6.11 ± 0.20 ^a^	6.46 ± 0.20 ^a^
18	3-Carene	−	−	−
19	Santolina triene	−	−	−
20	1,2-hexanediol-2-benzoate	−	−	−
21	3,7-Dimethyl-1,3,6-octatriene	−	−	−
22	3,7-Dimethyl-2,6-octadien-1-ol	−	−	−
23	Benzyl acetate	−	−	−
24	Lilac alcohol	−	−	−
25	Lilac aldehyde	−	−	−

I—Eastern Baltics (Russia); II—Moscow region (Russia); III—Minsk region (Belarus). “−”— not detected. Values in rows followed by the same letter do not differ significantly (*p* > 0.05), as assessed by the post hoc test (Tukey test). Data presented as a mean ± SD (n = 3).

**Table 3:** Assay of individual biologically active substances (content, mg/kg) in *D. maculata* samples from different regions.

Peak No.	Substances	Growing Region		
		I [7]	II	III
1	Rutin	4.54 ± 0.13 ^a^	3.46 ± 0.13 ^b^	4.12 ± 0.13 ^a^
2	Hyperoside	−	−	−
3	Ferulic acid	20.62 ± 0.61 ^a^	18.74 ± 0.61 ^b^	19.37 ± 0.61 ^ab^
4	Quercetin	10.73 ± 0.32 ^a^	9.94 ± 0.32 ^a^	10.18 ± 0.32 ^a^
5	Kaempferol	−	−	−
6	Disulfuretin	−	−	−
7	Sulfuretin	−	−	−
8	Sulfurein	−	−	−
9	Gallic acid	31.49 ± 0.94 ^a^	29.89 ± 0.94 ^b^	30.74 ± 0.94 ^ab^
10	Methyl gallate	−	−	−
11	Pentagalloyl glucose	−	−	−
12	3,3′,4′,5,6,7-hexahydroxyflavonone	12.87 ± 0.38 ^a^	10.39 ± 0.38 ^b^	12.06 ± 0.38 ^a^
13	3,3′,4′,5,5′,7-hexahydroxyflavonone	14.32 ± 0.42 ^a^	12.48 ± 0.42 ^b^	13.38 ± 0.42 ^b^
14	3-*О-*α-L-rhamnofuranoside	−	−	−
15	3,3′,4′,5,5′,7-hexahydroxyflavulium(1+)	−	−	−
16	7-*О*-β-D glucopyranoside	−	−	−
17	3,3′,4′,7-tetrahydroxyflavonone	−	−	−
18	3-Carene	−	−	−
19	Santolina triene	−	−	−
20	1,2-hexanediol-2-benzoate	−	−	−
21	3,7-Dimethyl-1,3,6-octatriene	−	−	−
22	3,7-Dimethyl-2,6-octadien-1-ol	−	−	−
23	Benzyl acetate	−	−	−
24	Lilac alcohol	−	−	−
25	Lilac aldehyde	−	−	−

I—Eastern Baltics (Russia); II—Moscow region (Russia); III—Minsk region (Belarus). “−”— not detected. Values in rows followed by the same letter do not differ significantly (*p* > 0.05), as assessed by the post hoc test (Tukey test). Data presented as a mean ± SD (n = 3).

**Table 4:** Assay of individual biologically active substances (content, mg/kg) in *P. chlorantha* samples from different regions.

Peak No.	Substances	Growing Region		
		I	II	III
1	Rutin	−	−	−
2	Hyperoside	−	−	−
3	Ferulic acid	−	−	−
4	Quercetin	−	−	−
5	Kaempferol	−	−	−
6	Disulfuretin	−	−	−
7	Sulfuretin	−	−	−
8	Sulfurein	−	−	−
9	Gallic acid	−	−	−
10	Methyl gallate	−	−	−
11	Pentagalloyl glucose	−	−	−
12	3,3′,4′,5,6,7-hexahydroxyflavonone	−	−	−
13	3,3′,4′,5,5′,7-hexahydroxyflavonone	−	−	−
14	3-*О-*α-L-rhamnofuranoside	−	−	−
15	3,3′,4′,5,5′,7-hexahydroxyflavulium(1+)	−	−	−
16	7-*О*-β-D glucopyranoside	−	−	−
17	3,3′,4′,7-tetrahydroxyflavonone	−	−	−
18	3-Carene	4.52 ± 0.13 ^a^	2.31 ± 0.13 ^b^	4.21 ± 0.13 ^a^
19	Santolina triene	6.71 ± 0.20 ^a^	5.64 ± 0.20 ^b^	6.42 ± 0.20 ^a^
20	1,2-hexanediol-2-benzoate	8.74 ± 0.26 ^a^	7.26 ± 0.26 ^b^	8.14 ± 0.26 ^s^
21	3,7-Dimethyl-1,3,6-octatriene	17.62 ± 0.52 ^a^	15.33 ± 0.52 ^b^	16.32 ± 0.52 ^c^
22	3,7-Dimethyl-2,6-octadien-1-ol	18.18 ± 0.54 ^a^	16.13 ± 0.54 ^b^	17.33 ± 0.54 ^a^
23	Benzyl acetate	14.37 ± 0.43 ^a^	13.16 ± 0.43 ^a^	13.53 ± 0.43 ^a^
24	Lilac alcohol	11.73 ± 0.35 ^a^	10.61 ± 0.35 ^a^	10.81 ± 0.35 ^a^
25	Lilac aldehyde	9.86 ± 0.29 ^a^	8.15 ± 0.29 ^b^	9.44 ± 0.29 ^a^

I—Eastern Baltics (Russia); II—Moscow region (Russia); III—Minsk region (Belarus). “−”— not detected. Values in rows followed by the same letter do not differ significantly (*p* > 0.05), as assessed by the post hoc test (Tukey test). Data presented as a mean ± SD (n = 3).

**Table 5:** Trace element composition (quantitative content, mg/kg of dry matter) of the studied medicinal plants from different regions.

Plant Samples	Growing Region	Elements					
		K	Na	Mg	Ca	S	P
*C* *. coggygria*	I	4630.2 ± 138.9 ^a^	9716.4 ± 291.5 ^a^	2077.2 ± 62.3 ^a^	3960.2 ± 118.8 ^a^	4580.5 ± 137.4 ^a^	1287.5 ± 38.6
	II	4247.4 ± 138.9 ^b^	9019.4 ± 291.5 ^b^	1758.2 ± 62.3 ^b^	3162.8 ± 118.8 ^b^	4136.3 ± 137.4 ^b^	986.9 ± 38.6 ^b^
	III	4448.5 ± 138.9 ^c^	9658.7 ± 291.5 ^a^	1975.3 ± 62.3 ^c^	3764.3 ± 118.8 ^c^	4279.8 ± 137.4 ^b^	1093.7 ± 38.6 ^b^
*D* *. maculate*	I	1173.7 ± 35.2 ^a^	9830.1 ± 295.0 ^a^	277.1 ± 8.3 ^a^	2070.0 ± 62.1 ^a^	5563.9 ± 166.9 ^a^	2410.8 ± 72.3 ^a^
	II	1043.6 ± 35.2 ^b^	9486.4 ± 295.0 ^b^	195.3 ± 8.3 ^b^	1869.1 ± 62.1 ^b^	4574.2 ± 166.9 ^b^	2106.3 ± 72.3 ^b^
	III	1101.9 ± 35.2 ^a^	9635.8 ± 295.0 ^c^	202.6 ± 8.3 ^b^	1998.6 ± 62.1 ^c^	5105.6 ± 166.9 ^c^	2190.9 ± 72.3 ^b^
*P* *. chlorantha*	I	2044.9 ± 61.3 ^a^	9633.5 ± 289.0 ^a^	429.5 ± 12.9 ^a^	3173.5 ± 95.2 ^a^	6240.8 ± 187.2 ^a^	759.3 ± 22.8 ^a^
	II	1874.1 ± 61.3 ^b^	9211.7 ± 289.0 ^b^	316.2 ± 12.9 ^b^	2950.5 ± 95.2 ^b^	5216.4 ± 187.2 ^b^	602.3 ± 22.8 ^b^
	III	1999.1 ± 61.3 ^a^	9483.2 ± 289.0 ^c^	398.5 ± 12.9 ^a^	3061.1 ± 95.2 ^a^	5879.5 ± 187.2 ^c^	694.7 ± 22.8 ^a^

I—Eastern Baltics (Russia); II—Moscow region (Russia); III—Minsk region (Belarus). Values in columns (for each plant species) followed by the same letter do not differ significantly (*p* > 0.05), as assessed by the post hoc test (Tukey test). Data presented as a mean ± SD (n = 3).

**Table 6:** The content of organic acids and vitamins (mg/kg) in *C. coggygria* samples from different regions.

Substances	Growing Region		
	I [8]	II	III
Succinic acid	577.7 ± 17.3 ^a^	501.1 ± 17.3 ^b^	527.9 ± 17.3
Benzoic acid	14.8 ± 0.4 ^a^	12.5 ± 0.4 ^b^	13.4 ± 0.4 ^b^
Fumaric acid	5.2 ± 0.2 ^a^	3.8 ± 0.2 ^b^	4.3 ± 0.2 ^ab^
Citric acid	816.8 ± 24.5 ^a^	748.3 ± 24.5 ^b^	793.6 ± 24.5 ^a^
Oxalic acid	119.1 ± 3.6 ^a^	102.6 ± 3.6 ^a^	110.0 ± 3.6 ^a^
Malic acid	1329.5 ± 39.9 ^a^	1002.3 ± 39.9 ^b^	1103.4 ± 39.9 ^c^
Ascorbic acid	72.12 ± 2.16 ^a^	50.18 ± 2.16 ^b^	63.77 ± 2.16 ^c^
Thiamine	3.30 ± 0.09 ^a^	2.00 ± 0.09 ^b^	2.93 ± 0.09 ^ab^
Riboflavin	2.14 ± 0.06 ^a^	1.04 ± 0.06 ^b^	1.94 ± 0.06 ^a^

I—Eastern Baltics (Russia); II—Moscow region (Russia); III—Minsk region (Belarus). Values in rows followed by the same letter do not differ significantly (*p* > 0.05), as assessed by the post hoc test (Tukey test). Data presented as a mean ± SD (n = 3).

**Table 7:** The content of organic acids and vitamins (mg/kg) in *D. maculata* samples from different regions.

Substances	Growing Region		
	I [7]	II	III
Succinic acid	365.6 ± 11.0 ^a^	285.3 ± 11.0 ^b^	321.3 ± 11.0 ^a^
Benzoic acid	36.5 ± 0.9 ^a^	21.8 ± 0.9 ^b^	29.7 ± 0.9 ^b^
Fumaric acid	7.4 ± 0.2 ^a^	5.8 ± 0.2 ^b^	6.8 ± 0.2 ^a^
Citric acid	3780.5 ± 113.4 ^a^	3081.2 ± 113.4 ^b^	3273.8 ± 113.4 ^b^
Oxalic acid	445.3 ± 13.3 ^a^	218.9 ± 13.3 ^b^	390.6 ± 13.3 ^a^
Malic acid	856.7 ± 25.7 ^a^	739.1 ± 25.7 ^b^	816.4 ± 25.7 ^a^
Ascorbic acid	49.63 ± 1.48 ^a^	27.93 ± 1.48 ^b^	41.66 ± 1.48 ^a^
Thiamine	2.05 ± 0.06 ^a^	1.07 ± 0.06 ^b^	1.82 ± 0.06 ^a^
Riboflavin	1.09 ± 0.03 ^a^	0.74 ± 0.03 ^a^	0.88 ± 0.03 ^a^

I—Eastern Baltics (Russia); II—Moscow region (Russia); III—Minsk region (Belarus). Values in rows followed by the same letter do not differ significantly (*p* > 0.05), as assessed by the post hoc test (Tukey test). Data presented as a mean ± SD (n = 3).

**Table 8:** The content of organic acids and vitamins (mg/kg) in *P. chlorantha* samples from different regions.

Substances	Growing Region		
	I [8]	II	III
Succinic acid	988.2 ± 29.6 ^a^	851.7 ± 29.6 ^b^	893.2 ± 29.6 ^c^
Benzoic acid	34.1 ± 1.0 ^a^	20.1 ± 1.0 ^b^	28.6 ± 1.0 ^c^
Fumaric acid	13.2 ± 0.4 ^a^	10.6 ± 0.4 ^b^	11.5 ± 0.4 ^b^
Citric acid	9898.8 ± 296.9 ^a^	9146.5 ± 296.9 ^b^	9318.2 ± 296.9 ^c^
Oxalic acid	569.7 ± 17.1 ^a^	407.2 ± 17.1 ^b^	485.6 ± 17.1 ^ab^
Malic acid	1119.4 ± 33.6 ^a^	958.1 ± 33.6 ^b^	1009.9 ± 33.6 ^b^
Ascorbic acid	18.44 ± 0.55 ^a^	15.39 ± 0.55 ^b^	17.00 ± 0.55 ^c^
Thiamine	1.19 ± 0.03 ^a^	0.89 ± 0.03 ^a^	0.95 ± 0.03 ^a^
Riboflavin	0.86 ± 0.02 ^a^	0.61 ± 0.02 ^a^	0.71 ± 0.02 ^a^

I—Eastern Baltics (Russia); II—Moscow region (Russia); III—Minsk region (Belarus). Values in rows followed by the same letter do not differ significantly (*p* > 0.05), as assessed by the post hoc test (Tukey test). Data presented as a mean ± SD (n = 3).

**Table 9:** Antimicrobial (diameter of the lysis zone, mm) and antioxidant activity of medicinal plants from different regions.

Plant Samples	Growing Region	Test Cultures					Antioxidant Activity, Mg AA/g
		1	2	3	4	5	
*C*. *coggygria*	I [8]	16.5 ± 0.5 ^a^	14.5 ± 0.5 ^a^	11.0 ± 0.5 ^a^	13.0 ± 0.5 ^a^	14.5 ± 0.5 ^a^	145.09 ± 7.25 ^a^
	II	13.6 ± 0.5 ^b^	12.5 ± 0.5 ^b^	9.0 ± 0.5 ^b^	11.6 ± 0.5 ^b^	12.2 ± 0.5 ^b^	107.29 ± 7.25 ^b^
	III	15.2 ± 0.5 ^a^	13.7 ± 0.5 ^ab^	10.3 ± 0.5 ^a^	12.4 ± 0.5 ^b^	13.5 ± 0.5 ^a^	110.35 ± 7.25 ^b^
*D*. *maculate*	I [7]	17.0 ± 0.5 ^a^	15.0 ± 0.5 ^a^	14.0 ± 0.5 ^a^	15.0 ± 0.5 ^a^	17.0 ± 0.5 ^a^	217.89 ± 10.89 ^a^
	II	15.2 ± 0.5 ^b^	14.3 ± 0.5 ^a^	12.4 ± 0.5 ^b^	13.0 ± 0.5 ^b^	15.4 ± 0.5 ^b^	193.75 ± 10.89 ^b^
	III	16.4 ± 0.5 ^a^	14.8 ± 0.5 ^a^	13.0 ± 0.5 ^ab^	14.0 ± 0.5 ^ab^	16.1 ± 0.5 ^ab^	201.27 ± 10.89 ^ab^
*P*. *chlorantha*	I	17.0 ± 0.5 ^a^	18.0 ± 0.5 ^a^	16.5 ± 0.5 ^a^	17.5 ± 0.5 ^a^	18.0 ± 0.5 ^a^	220.43 ± 11.02 ^a^
	II	15.1 ± 0.5 ^b^	15.9 ± 0.5 ^b^	14.6 ± 0.5 ^b^	15.6 ± 0.5 ^b^	16.8 ± 0.5 ^b^	199.85 ± 11.02 ^b^
	III	16.2 ± 0.5 ^ab^	17.0 ± 0.5 ^ab^	15.8 ± 0.5 ^a^	16.2 ± 0.5 ^b^	17.7 ± 0.5 ^ab^	209.86 ± 11.02 ^ab^

I—Eastern Baltics (Russia); II—Moscow region (Russia); III—Minsk region (Belarus). 1—*E. coli*; 2—*S. aureus*; 3—*P. vulgaris*; 4—*C. albicans*; 5—*L. mesenteroides*. Values in columns (for each plant species) followed by the same letter do not differ significantly (*p* > 0.05), as assessed by the post hoc test (Tukey test). Data presented as a mean ± SD (n = 3).

**Figure A1:**
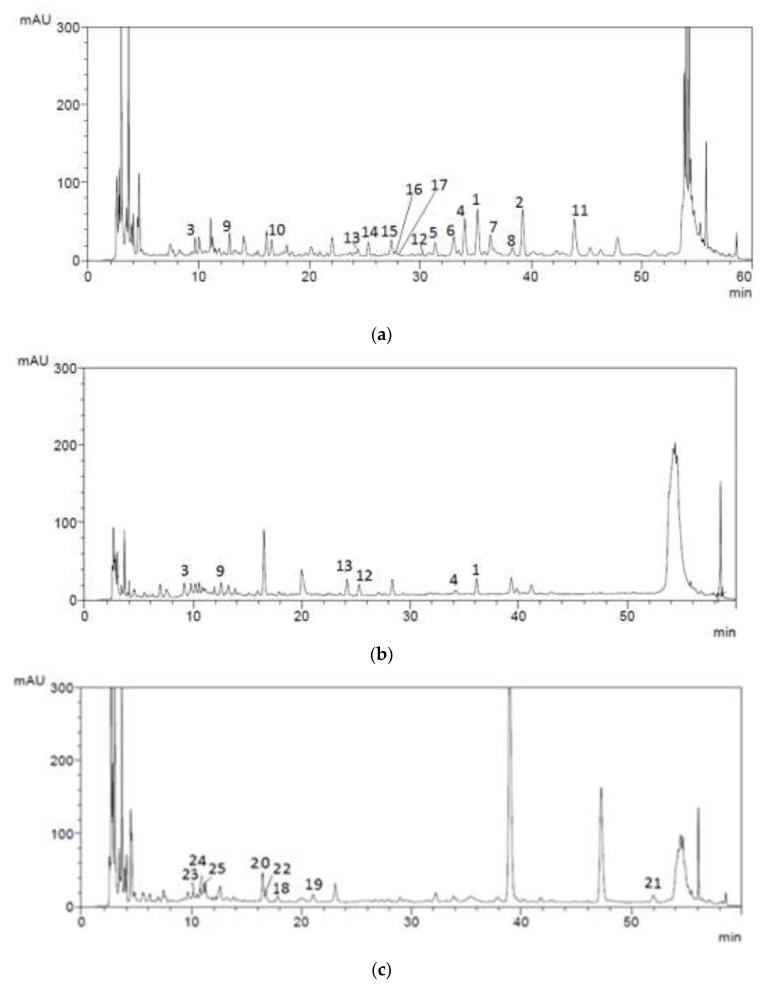
Chromatographic profile of the chemical composition of a plant (**a**) *Cotinus coggygria*, (**b**) *Dactylorhiza maculata*, (**c**) *Platanthera chlorantha*: 1—rutin, 2—Hyperoside, 3—ferulic acid, 4—quercetin, 5—kaempferol, 6—disulfuretin, 7—sulfuretin, 8—sulfurein, 9—gallic acid, 10—methyl gallate, 11—pentagalloyl glucose, 12—3,3′,4′,5,6,7-hexahydroxyflavonone, 13—3,3′,4′,5,5′,7-hexahydroxyflavonone, 14—3-*О-*α-L-rhamnofuranoside, 15—3,3′,4′,5,5′,7-hexahydroxyflavulium, 16—7-*О*-β-D glucopyranoside, 17—3,3′,4′,7-tetrahydroxyflavonone, 18—3-Carene, 19—santolina triene, 20—1,2-hexanediol-2-benzoate, 21—3,7-dimethyl-1,3,6-octatriene, 22—3,7-dimethyl-2,6-octadien-1-ol, 23—benzyl acetate, 24—lilac alcohol, 25—lilac aldehyde.

## Data Availability

Data are contained within the article.
